# T cell aging and Alzheimer’s disease

**DOI:** 10.3389/fimmu.2023.1154699

**Published:** 2023-04-04

**Authors:** Lin Guo, Xiaoting Li, Timothy Gould, Zhan-You Wang, Wenqiang Cao

**Affiliations:** ^1^ Key Laboratory of Major Chronic Diseases of Nervous System of Liaoning Province, Health Sciences Institute of China Medical University, Shenyang, China; ^2^ Department of Rehabilitation, Shengjing Hospital of China Medical University, Shenyang, China; ^3^ Rubedo Life Sciences, Sunnyvale, CA, United States

**Keywords:** T cell aging, Alzheimer’s disease (AD), thymic involution, senescence, neuroinflammation

## Abstract

The brain has long been considered an immune-privileged organ due to the presence of the blood-brain barrier (BBB). However, recent discoveries have revealed the underestimated role of T cells in the brain through the meningeal lymphatic system. Age is the primary risk factor for Alzheimer’s disease (AD), resulting in marked age-dependent changes in T cells. Manipulating peripheral T cell immune response has been shown to impact AD, but the relationship between T cell aging and AD remains poorly understood. Given the limited success of targeting amyloid beta (Aβ) and the growing evidence of T cells’ involvement in non-lymphoid organ aging, a deeper understanding of the relationship between T cells and AD in the context of aging is crucial for advancing therapeutic progress. In this review, we comprehensively examine existing studies on T cells and AD and offer an integrated perspective on their interconnections in the context of aging. This understanding can inform the development of new interventions to prevent or treat AD.

## Introduction

**Figure 1:**
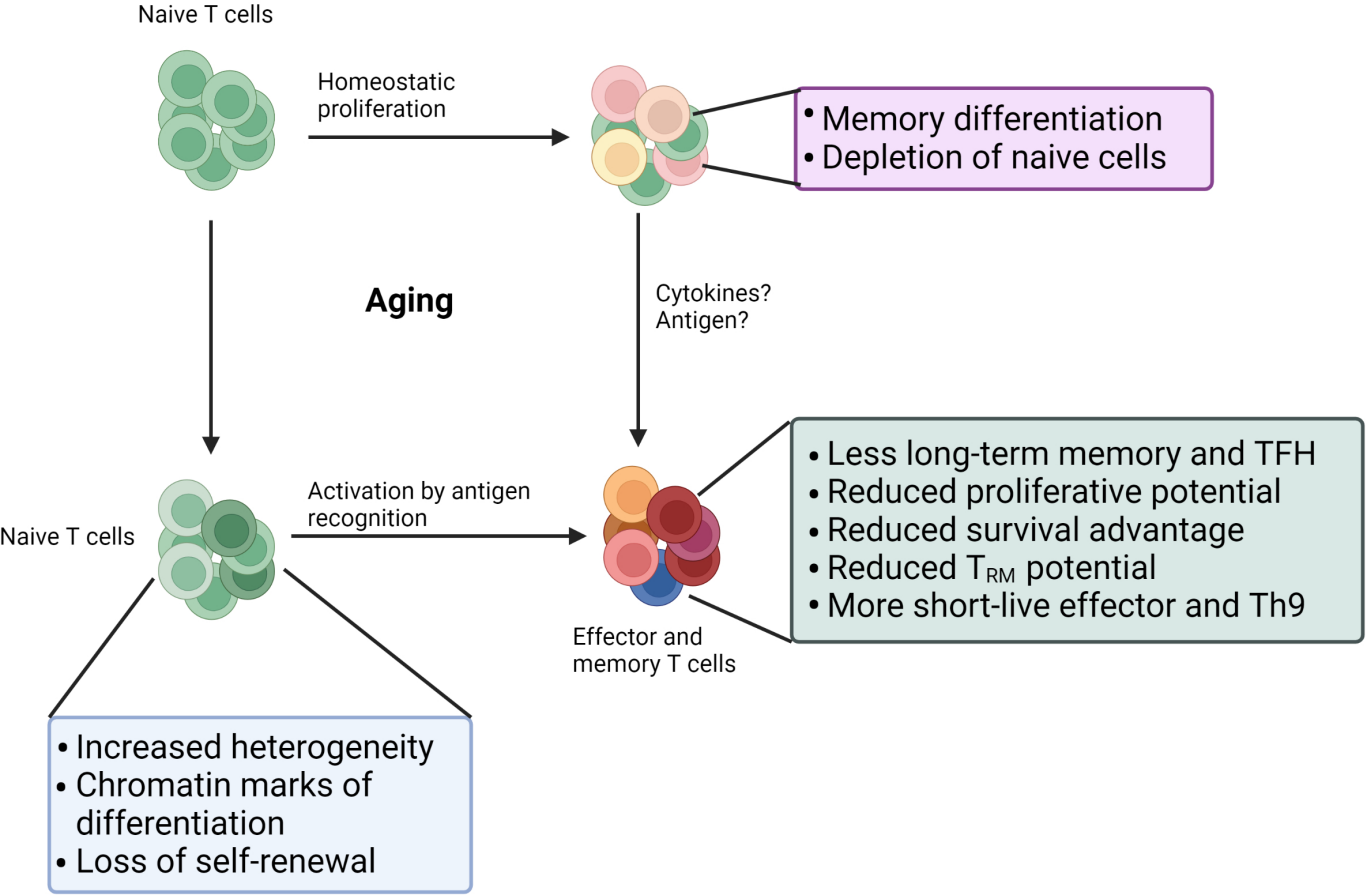
Age-related changes in the T cell. Self-renewal of naïve T cell cells via homeostatic proliferation often fails in old individuals: 1) memory differentiation driven by homeostatic cytokines; 2) death due to lack of survival signal. Age also leads to significant changes in naïve T cell compartment: more differentiated states, increased heterogeneity, and reduced quiescence. Those changes in naïve T cells combined with changes in activation stages result in dysfunctions in aged effector or memory T cells. Furthermore, age impairs the function of existing memory.

T cell aging begins with thymic involution, one of the most well-studied hallmarks of immune aging, and occurs during childhood and puberty (1). As a result, the thymus significantly reduces its production of new naïve T cells, and the maintenance of peripheral naïve T cells heavily relies on homeostatic proliferation. Although the homeostatic proliferation of T cells can replenish the T cell compartment, the number of naïve T cells still declines significantly with age, especially for CD8 T cells (2, 3). The inability to maintain naivety leads to failure of self-renewal of naïve T cells via hemostatic proliferation in old individuals, which occurs more often within CD8 T cells than within CD4 T cells. In line with this difference, terminally differentiated T effector memory CD45RA+ (TEMRA) cells, which display senescent characteristics, accumulate more among CD8 T cells than among CD4 T cells in old individuals (4). Interestingly, virtual memory cells in humans that develop features of cellular senescence accumulate with age and are found among TEMRA cells (5, 6), suggesting generation of TEMRA cells partially comes from cytokines stimulation. Although fewer TEMRA cells exist among CD4 T cells, aged CD4 T cells also display powerful pro-inflammatory or anti-inflammatory features, which closely link to disorders in old individuals (7, 8). Besides, the long-term memory and T follicular helper (Tfh) cell development potential of aged naïve T cells is impaired due to upregulated CD39, miR-21 expression, and impaired proteostasis (9–12), suggesting that age-related changes already exist at the naïve stage. Indeed, single-cell RNA sequencing of naïve CD4 T cells revealed a distinct profile of aged naïve T cells (13). Furthermore, the functionality of aged memory T cells is still inferior due to decreased CD73+ memory cells (14). Thus, age-related alterations in naïve cells, memory cells, homeostatic proliferation, and activation shape significantly affect the function of T cells in old individuals (**Figure 1**).

Alzheimer’s disease (AD) is an age-related neurodegenerative brain disorder and is the most prevalent neurodegenerative disease in the aged population (15). Its cardinal characteristics are the extracellular deposit of amyloid fibrillary amyloid beta (Aβ) and neurofibrillary tangles mediated by aggregated hyperphosphorylated tau protein (16, 17). Several therapeutic drugs have been developed, and many clinical trials have been conducted based on the amyloid cascade hypothesis. However, most AD-related clinic trials have failed, necessitating that promote researchers revisit the mechanisms underlying AD (18).

An increasing body of evidence demonstrates that dysfunctional neuroinflammation and systemic inflammation are found in AD and associated with clinical indications (19). Indeed, Yousefzadeh et al. found that an aged immune system can drive the senescence of solid organs, including the brain (20). Accumulation of senescent cells in the brain has been reported to worsen pathologic impairments in AD mice models (21, 22). Hence, the aged immune system is supposed to exacerbate the pathologic process of AD. It raises a question: as a critical part of the immune system, what is the role of aged T cells in the senescence of non-immune organs? A recent study revealed that Tfam deficiency in T cells induced inflammaging, which led to multimorbidity and premature senescence, including neurologic impairments (23). Although the authors did not evaluate the AD-specific alterations, neurodegenerative changes were observed (23). Tfam-deficient T cells exhibit some identical features as aged T cells; arguably, T cell aging plays an essential role in AD. A recent report supports it: the frequency of TEMRA CD8 T cell—its accumulation is one of the hallmarks of T cell aging–in peripheral and cerebrospinal fluid (CSF) increases in AD patients. It is negatively correlated with cognition (24). This review summarizes the findings of such age-related changes and the potential role of T cell aging in AD.

## Thymic involution and AD

One of the primary characteristics of T cell aging is thymic involution, which results in reduced generation of naïve T cells (25). In humans, the thymus is a primary source of T cells before puberty. However, its contribution decreases to less than 20% by early adulthood and further declines with age due to thymic involution. Thymic involution in mice is more gradual than in humans, and thymic output still plays a role in maintaining naïve T cells even in aged mice (26–28). Thymic involution directs the senescence of cells through three aspects: 1) impaired negative selection, 2) imbalanced Treg generation, and 3) reduced thymopoiesis (29).

Impaired negative selection in the aged thymus leads to an increased generation of self-reactive T cells (29). Aβ-specific T cells increase in AD patients and old individuals (30), which can infiltrate the brain parenchyma and promote AD in mice (31). Whether the increased Aβ-specific T cells in AD patients and old individuals is a consequence of thymic involution remains elusive. Thymic involution also dramatically changes Treg generation. Due to reduced TCR signaling strength in the atrophied thymus, the generation of tTreg (thymic Treg) cells increased (32), which has been shown to play an important role in AD. Depending on their location, Treg cells’ role in AD is protective or pathogenic (33). Treg cells residing in the choroid plexus (CP) inhibit CP leukocyte trafficking via IL-10, which is detrimental to AD (34, 35). However, Treg cells in the brain inhibit neuroinflammation and could ameliorate the pathology of AD (36). Based on these facts, increased Aβ-specific T cells and generation of Treg cells in aged thymus probably promote AD. The following two sections will discuss reduced thymopoiesis-mediated decreased naïve T cells and increased senescent T cells.

Age-related thymic involution is mainly driven by increased sex steroids, mediated by steroid signaling in thymic epithelial cells (TEC) (37). Surgical or chemical sex steroid ablation improves thymic rejuvenation. In addition to age, specific infection and physiological changes, such as schistosomiasis and pregnancy, induce acute thymic involution (38, 39), which affect the process of infection and pregnancy through T cell response. Whether changes in AD influence thymic involution remains an interesting question. As we know, AD is also associated with age-related sex hormone fluctuations, but the effect of sex steroids on AD is controversial. Numerous epidemiologic studies suggest a protective role for estrogen in Alzheimer’s disease (40, 41). However, a recent study indicates that long-term hormone replacement therapy (HRT) is associated with an increased risk of AD in postmenopausal women (42, 43). Long-term treatment with hormones, especially at an earlier age, accelerates thymic involution, which is probably detrimental to AD. Consistently, Leuprorelin, a luteinizing hormone-releasing hormone (LHRH) agonist, inhibits sex steroids generation, rejuvenates T cell immunity via increased thymic activity (44), and also has been found to ameliorate neuropathologic processes in APP/PS1 mice and delay loss of cognition in a subgroup of AD patients (45, 46). Another piece of evidence comes from the study of congenital heart disease (CHD); the thymus is removed during cardiac surgery in newborns with CHD, leading to T cell aging (47). Adults with CHD have an increased risk of developing AD (48), which is probably associated with thymectomy. Further investigations are warranted to explore the potential relationship between AD and thymic activity.

## Naïve/memory T cells imbalance and AD

As described in the previous section, due to reduced thymic output, naïve/memory T cell imbalance is the most significant hallmark of T cell aging. Although homeostatic proliferation can considerably compensate naïve T cells in young individuals, self-renewal via homeostatic proliferation often fails in old individuals (49). Homeostatic proliferation depends on signaling from IL7 and MHC/self-antigen (50). Decreased IL-7 receptor expression on aged T cells and less accessibility to secondary lymph organs contribute to the loss of naïve T cells with age (51, 52). Besides, due to declined TRIB2, aged naïve T cells are prone to differentiation towards memory response to homeostatic cytokines that further contribute to the loss of naïve T cells (53).

Recent studies have shown that naïve T cells are more heterogeneous than previously thought (54)—aging shapes dramatically naïve T cells’ epigenetic landscape (55–57). Age-related changes in naïve T cell chromatin accessibility are similar to those in activated and differentiated T cells, and this phenomenon is more pronounced in naïve CD8 T cells (58–60). At the single-cell level, aging increases heterogeneity in gene expression during early activation, indicating diversity in differentiation states (13, 61). Age-associated transcriptional factors and microRNA network changes account for those differentiation states (9, 13, 60, 62–64). A phenotypically unique subset of “young naïve T cells” –CD45RA^high^CD27^high^CD38^+^CD25^neg^–is lost with age (65). More differentiated states in aged naïve T cells imply less effector plasticity. Increased transforming growth factor-β receptor 3 and CD39 in aged naïve CD4 T cells favor Th9 and inhibit Tfh differentiation, respectively (11, 66). Moreover, due to increased miR-21, naïve CD4 T cells from old individuals are prone to differentiate into inflammatory effector cells rather than Tfh and memory precursor cells (9). In addition to the frequency of naïve T cells, aging changes dramatically the states of naïve T cells.

Peripheral T cells in AD patients and mouse models have been studied widely (67), and a decreased ratio of naïve to memory T cells is a consistent phenotype. Tau and Aβ have been reported to trigger T cell-specific response (30, 68), suggesting that peripheral tau and Aβ protein mimic “infection” and drive naïve T cell differentiation toward memory cells. However, the explanation is likely more complex. Substantial clonally expanded CD8 T cells in AD patients specifically recognize antigens from Epstein-Barr Virus (EBV), not AD-related proteins (24). Hence, other factors contribute to the loss or differentiation of naïve T cells in AD. Studies from atherosclerosis, another age-related disease, show that accumulation of cholesterol in T cells induces T cell aging phenotype, including less frequent naïve T cells (69). Interestingly, CD8 virtual memory T cells accumulate with age and upregulate lipid rafts (6, 70). Dysfunction of lipid metabolism in AD is associated with lipid raft changes and activation of innate immune cells (71), suggesting that lipid metabolism dysfunction in AD possibly accelerates differentiation of naïve T cells via elevated cholesterol or lipid rafts.

Poor naivety maintenance of T cells in AD may contribute to impaired cognition, and age-related downregulation of the naïve marker CCR7 on T cells is associated with worsened cognition (72). Furthermore, CCR7-deficiency exacerbates brain Aβ deposition and cognitive decline in 5xFAD mice, and anti-CD25 treatment improves cognition (72). Although the authors attributed it to the depletion of Treg cells and did not evaluate the states of naïve T cells with anti-CD25 injection, recently Zhang et al. showed that Helios downregulated CD25 and prevented an effector cell response, anti-CD25 treatment shifted the chromatin accessibility states toward those of younger individuals and away from an effector memory pattern (13). Hence, in addition to the depletion of Treg, CD25 inhibition probably also influences pathologies of AD via maintaining the naïve pattern of T cells. More specific inventions are required to investigate the impact of naïve cells on AD. TRIB2 is a vital regulator of naivety, and its stabilizing drugs have been applied to clinical treatment (53). It is thus of great interest to evaluate the effect of TRIB2-mediated maintenance of naïve T cells to AD.

## Senescent T cells and AD

Aging leads to the accumulation of senescent cells, including senescent T cells, which share many standard features of senescence with other senescent cells, including short telomeres and reduced telomerase activity, DNA damage, mitochondrial dysfunction, and β-galactosidase activity (73). Functionally, senescent T cells are defective in their TCR-induced proliferation and secrete abundant inflammatory factors—as part of the SASP (senescence-associated secretory phenotype). They lack the costimulatory receptors CD27 and CD28 and upregulate natural killer cell (NK)-related markers (74). Human TEMRA cells, which display this characteristic of cellular senescence and are associated with increased p38 signaling (4), are a primary subset of senescent T cells. Senescent T cell accumulation is likely a result of lifelong antigen stimulation, especially chronic virus infection (75). However, virtual memory T cells, driven by cytokines, also accumulate with age and exhibit similar features of senescence (6). CD4 T cells also acquire NK receptors and become cytotoxic with age, possibly associated with downregulated ThPOK (7, 76, 77). ThPOK expression declines in aged CD4 T cells (53). Thus, ThPOK may play a crucial role in accumulating senescent T cells.

CD8 TEMRA cells are found to increase in peripheral blood, and cerebrospinal fluid of AD patients and are correlated with impaired cognition (24). CD8 T cells from human brains (including AD patients) lack CD27 and CD28 expression, which are characteristics of TEMRA cells (78). Surprisingly, TCR-sequencing reveals that a high proportion of clonally expanded CD8 T cells among these TEMRA cells recognize EBV antigen (24), suggesting that these TEMRA cells probably play a role in AD via a bystander function. Why these TEMRA cells increase in AD patients is unclear. It is speculated that these TEMRA cells generate in the periphery via antigen or cytokine stimulation and migrate into the brain. Further investigation is needed to understand the mechanisms underlying peripheral alterations of AD associated with TEMRA cell accumulation.

CD8 TEMRA cells upregulate NK markers, which mediate the killing of senescent cells independent of TCR recognition (79). Increased frequency of CD8 TEMRA cells is supposed to enhance the clearance of senescent cells in the brain. However, intracerebral senescent cells accumulate in AD mice, which negatively affects the process of AD (21, 22), suggesting that the cytotoxicity of CD8 TEMRA cells in AD is impaired. Similarly, the ability of aged CD8 T cells to clear senescent cells is diminished due to elevated NKG2A and PD-1 and reduced binding of perforin (79–82). Furthermore, the low cytotoxicity of CD8 T cells is likely brain-specific. Compared to circulating CD8 T cells, CD8 T cells from human brains express less GZMB and perforin (78). Hence, reduced immune surveillance of senescent cells in the brain by CD8 T cells may contribute to AD development.

On the other hand, GZMK and TNFα secreted from senescent CD8 T cells promote the senescence of other cells, including brain cells, which probably exacerbate the accumulation of senescent cells in the brain (8, 23). Hence, the role of CD8 T cells in AD is complex (83, 84). Clearance of senescent cells in the brain requires functionally cytotoxic CD8 T cells. However, aged CD8 T cells are unable to kill senescent cells and induce more cellular senescence in the brain via SASP signals. In addition to the contribution of cellular senescence in the brain, the accumulation of aged CD8 T cells in the brain directly promotes axon and myelin degeneration via GzmB and IFNγ-mediated microglia activation (85, 86). These emphasize the importance of correcting the dysfunction of aged CD8 T cells, not simply via depletion or supplementation.

Senescent CD4 T cells acquire extreme pro-inflammatory and anti-inflammatory phenotypes with reduced effector plasticity. Peripheral CD4 T cells from AD patients exhibit a bias differentiation as seen in old individuals with increased activity of Th17, Th9, and Th1 (87–89). These T cell subsets can infiltrate the brain and be involved in the pathology of AD (31, 90, 91). IL-17 has been reported to trigger the onset of cognitive and synaptic deficits and the breakdown of the brain blood barrier, and blockade of IL-17 decreased cognitive impairments (92–94). The role of Th1 in AD is controversial (95). Intraventricular injection of Th1 cells favored microglia activation and clearance of Aβ (96, 97). However, when transferred peripherally, Th1 cells increased Aβ plaque burden (31). Although senescent T cells contribute to inflammaging and are detrimental in age-related disorders by producing pro-inflammatory cytokines, activating T cell responses by blocking PD-1 or transient depletion of peripheral Treg cells ameliorates disease pathology in mouse models of AD (34, 98). PD-1^+^ T cells and Treg cells accumulate in the periphery with aging in both mice and humans (99, 100), and PD-1 blockade and depletion of Treg cells appear to correct some age-related dysfunctions. These suggest that restoring the function of senescent T cells in the periphery could ameliorate AD pathology.

## Concluding remarks

Numerous poor outcomes from clinical trials on AD have stimulated the revisiting of AD etiology. Treatments targeting Aβ have shown discouraged outcomes in AD patients, suggesting that clearing Aβ alone is insufficient to delay the AD process. The role of Aβ in AD needs to be revisited in the context of new emerging hypotheses, such as senescence cascade and neuroinflammation, which are closely linked to T cells. Aging is the most significant risk factor for AD, dramatically shaping T cells’ function. Recent evidence shows that the immune system, especially T cells, plays a central role in whole-body senescence, highlighting the relevance of T cells in AD.

**Figure 2:**
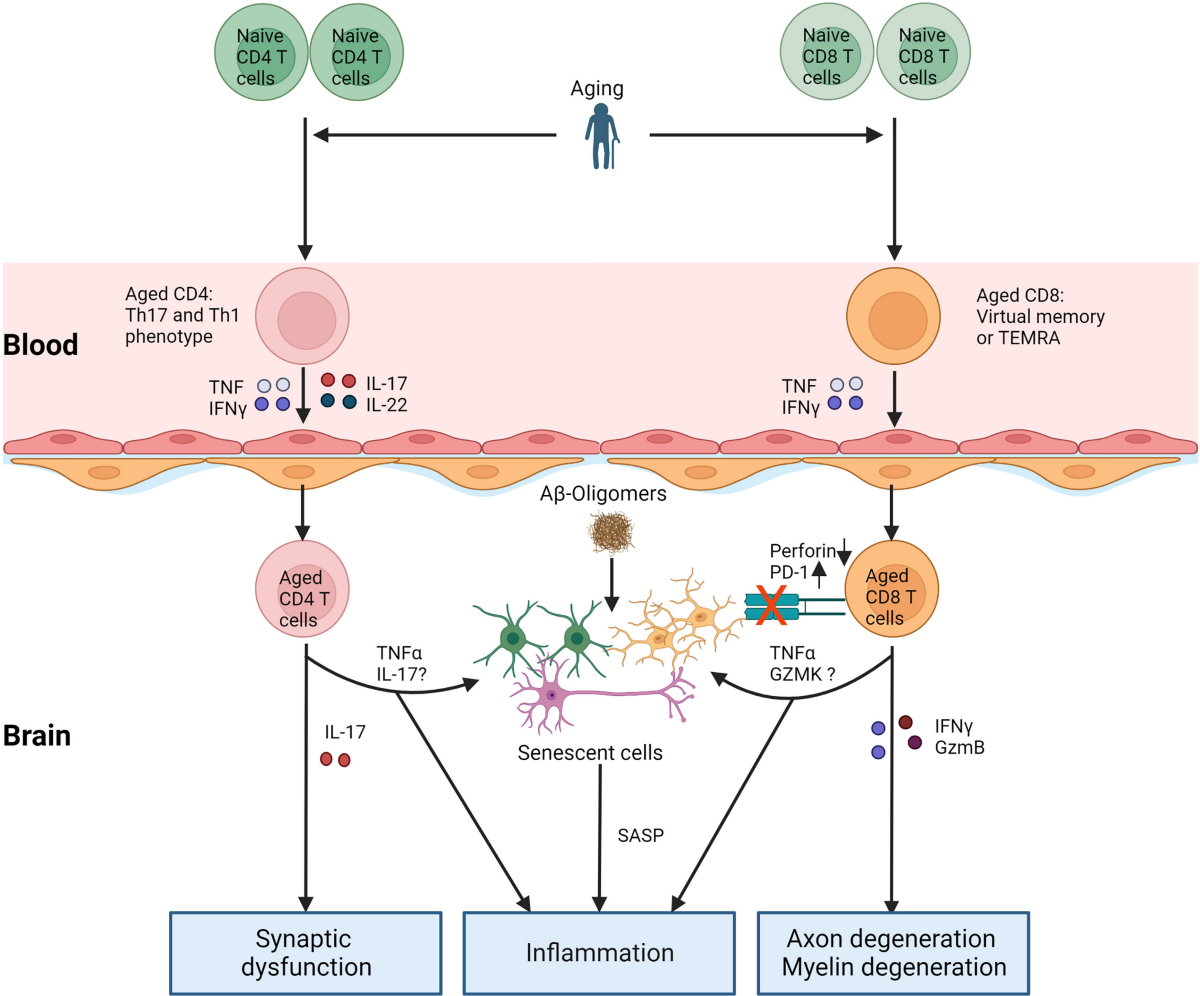
T cell aging and AD. With age, the number of naïve T cells decreases, and relative memory T cells (Th1 and Th17 for CD4 T cells, TEMRA, or virtual memory for CD8 T cells) accumulate. Cytokines secreted by aged T cells increase the permeability of BBB, which increases other immune cells or their entry into the brain. On the one hand, aged T cells in the brain directly contribute to neuroinflammation and damage to the nervous system by releasing cytokines. Aged T cells in the brain worsen the pathology of AD via the accumulation of senescent cells. TNFα, IL-17, and GZMK from aged T cells probably induce senescence in brain cells. Furthermore, increased PD-1 or decreased perforin in aged CD8 T cells in the brain inhibits the clearance of senescent cells. Aβ: amyloid β, TEMRA: T effector memory CD45RA+. SASP: senescence-associated secretory phenotype.

Current studies about T cell aging and AD are limited and indirect. We summarize the potential relationship between T cell aging and AD (**Figure 2**) and critical questions to warrant further investigation: 1) What is the role of intracerebral CD8 TEMRA cells in AD? Why cannot they kill accumulated senescent cells? 2) Whether induction of T cell aging, for example, thymectomy, exacerbates the pathology of AD, and what are the mechanisms? 3) How do changes in AD influence T cell aging? Answering these questions will deepen our understanding of the etiology of AD and the mechanism of T cell aging and open new possibilities by developing drugs to treat AD via targeting aged T cells.

## Author contributions

LG and XL prepared the manuscript. TG reviewed the manuscript. ZYW and WC edited and finalized the manuscript. All authors contributed to the article and approved the submitted version.
